# Supplementary material for: Are carbon emissions trading and green financial instruments synergistic? -Comprehensive quantitative research based on content analysis
Source: PLoS One. 2024 Mar 7;19(3):e0298601. doi: 10.1371/journal.pone.0298601 (PMC10919601; doi:10.1371/journal.pone.0298601)
Supplement: S1 Data — (ZIP) [file pone.0298601.s001.zip › Data/地级市Z_F工作报告.zip!/░▓╗╒/╗╞╔╜╩╨/└┤╫╘╣1⁄2╓┌║┼í╛┬φ┐╦╩2╛▌═°í┐_2012.docx]

各位代表：
        现在，我代表市人民政府，向大会作工作报告，请予审议，并请市政协委员和其他列席人员提出意见。

**一、2011年工作回顾**

        刚刚过去的一年，全市上下在省委、省政府和市委的坚强领导下，深入贯彻落实科学发展观，认真贯彻党的十七大、十七届五中、六中全会和省第九次党代会、市第五次党代会精神，紧紧围绕建设现代国际旅游城市这一战略目标，强力推进“十大工程”和“四区”建设，团结拼搏，锐意进取，经济社会发展呈现速度较快、结构优化、效益提升、民生改善的良好态势，实现了“十二五”的精彩起步、强势开局。
        **（一）各项目标全面完成。**去年是省政府对我市实施新的目标管理考核的第一年。我们严格按照省委、省政府的要求，持续推进转型发展、开放发展、绿色发展、和谐发展，圆满完成了省政府和市五届人大六次会议确定的各项目标任务。初步核算，全市实现地区生产总值378.8亿元、同比增长12.5%；财政收入64亿元、两年翻了一番多，增长44.6%、增速位居全省第三；固定资产投资370亿元、增长25.6%；社会消费品零售总额148.3亿元、增长17.9%；城镇居民人均可支配收入18669元、农民人均纯收入7952元，均高于全省平均水平，分别增长17.9%和18.4%。三次产业协调发展。农业增加值45.1亿元、增长4.4%，茶业综合产值61亿元、增长32.2%。规上工业增加值114亿元、增长24.7%，增速位居全省第三；完成工业技改投资42.1亿元、增长26.5%，高新技术企业达43家。服务业增加值158.2亿元、增长9.4%；全年接待游客3054.4万人次，旅游总收入251亿元，分别增长20%和24.2%，其中，入境游客131.4万人次，创汇3.85亿美元，分别增长25.1%和27.9%。民生工程、保障性住房建设、节能减排等省政府考核指标全面完成。
        **（二）重点工作扎实推进。**“十大工程”建设力度进一步加大，完成投资383.1亿元、增长22.5%。新安江延伸段综合开发一期工程基本完成，黄绩高速竣工，黄祁高速路基贯通，徽州500千伏输变电等一批重点工程建成使用。新型城乡规划体系初步形成，“十二五”专项规划编制工作全面完成。十五个重要节点快速推进，“四大整治”深入开展，关键区域控制进一步强化，土地矿产卫片执法检查实现“零约谈、零问责”目标。完成“百村千幢”25个古村落、268幢古民居的保护利用，绿色质量提升行动建成提升点532个，百佳摄影点加快建设，成为旅游文化新亮点。国家服务业综合改革试点全面推开，排定亿元以上重大项目240个、总投资2700亿元，徽州府衙修复等项目基本完成，花山谜窟激光秀、元一全景剧院、宏村阿菊、秀里影视村、黄山南大门环境综合整治、“智慧黄山”等项目扎实推进，西递、宏村跻身5A景区行列，黄山风景区成为世界自然保护联盟首个中国旅游区，入选全国低碳旅游实验区。新安江流域综合治理全面展开，全国首个跨省跨流域的新安江流域生态补偿机制试点顺利实施，总投资11.6亿元的61个试点项目启动建设，横江生态修复和丰乐河生态治理成效显著，沿线乡村生活垃圾处置体系基本建成。
        **（三）城乡面貌焕然一新。**中心城区完成投资116亿元、增长32.4%。屯光大道、齐云大道、西海路、北海路改造全面完成，梅林大桥基本竣工，花山大桥快速推进，文峰桥开工建设，城市道路交通网络进一步完善。市图书馆、档案馆、城市展示馆、老年大学和老干部活动中心、残疾人服务中心开工建设，新增和延伸公交线路5条，更新公交车47辆。“三小”改造年度任务全部完成。高铁新区“一场一河两区四桥七路”建设全面拉开，完成投资10.3亿元，安置区一期基本建成。徽州文化艺术长廊项目安置区、主干道主体工程完成，百师宫样板街、水景公园基本建成，全年完成投资8亿元。市经济开发区完成投资28亿元，梅林大道改造提升工程加快推进。城乡建设日新月异，城镇化率提高到45.1%。屯溪江南新城、黄山区城市道路路网完善、徽州区新四军军部旧址及周边环境整治、歙县慈张线拓宽改造、休宁县齐云大道三期、黟县城东城南新区、祁门新城区等城镇建设项目快速推进，区县城建设完成投资108亿元、增长35.3%；歙县蝉联全国文明县城。建制镇建设完成投资61亿元、增长37.9%；新农村建设完成投资30亿元，又有50个村达到“十有”标准。农村清洁工程加快推进，新增国家级生态乡镇4个、国家级生态村2个。我市荣获中国最佳休闲城市称号，在中部六省率先摘取中国人居环境奖城市的桂冠。
        **（四）人民生活持续改善。**扎实开展“七保”工作，精心组织实施36项民生工程，共投入资金15.4亿元，惠及140万城乡居民，继续保持全省前列。新增城镇就业3.84万人，下岗失业人员和就业困难人员再就业2.1万人，城镇登记失业率控制在3.9%以内。在全省率先实现城乡居民养老保险全覆盖，惠及城乡居民80.98万人、参保率94.2%，为17.6万符合领取条件人员发放基础养老金7639万元、发放率达100%。城镇职工医疗保险实现市级统筹，五项社会保险参保人数达83.7万人。稳步提高企业离退休人员养老金、城乡低保、重点优抚和五保对象补助标准，发放城乡居民大病救助资助金2400万元。新改扩建农村敬老院10所，新建留守儿童之家193个。及时调整全市最低工资标准，清欠农民工工资5716万元。建立社会救助和保障标准与物价上涨挂钩联动机制，发放价格临时补贴6301万元，投入菜篮子建设资金4200万元，新建蔬菜基地8000亩，新建改建中心城区农贸市场3个。投入资金6459.2万元，改造农村危房8074户。“一卡通”发放涉农补贴4.8亿元。全力开展抗洪救灾，11.2万受灾群众得到妥善安置。完成44个地质灾害隐患点避让搬迁和工程治理，所有区县均获得地质灾害群测群防“十有县”称号。解决9.4万农村人口饮水安全问题。深入开展整村推进扶贫工程，5000人实现稳定脱贫。
        **（五）改革开放成效显著。**启动事业单位分类改革，基层医药卫生体制改革、农村综合改革继续深化，“一事一议”财政奖补试点取得成效，我市荣获“全国文化体制改革工作先进地区”称号。组建市创业风险投资有限公司，黄山江南林权交易所挂牌成立。持续超常规推进金融发展与服务，新设立农村商业银行1家、村镇银行2家、小额贷款公司7家、亿元以上融资性担保公司3家，黄山旅游集团2亿元中期债券成功发行；落实银企对接资金97.5亿元，金融机构年末人民币贷款余额348.99亿元，增长24.2%。全省服务业发展大会在我市成功召开，成功举办黄山国际旅游节暨徽文化节、首届新安江龙舟赛、黄山茶叶暨名优农产品博览会等重大活动，精心组织参加皖台、皖粤经贸合作和徽商大会、厦洽会、中博会等系列招商活动。黄山至首尔定期航班开通，黄山至台湾直航班机将于18日首航，黄山机场全年旅客吞吐量首次突破50万人次。全省首家外资旅行社挂牌运营，大型连锁超市大润发建成营业。全年招商引资实际到位内资375.3亿元，利用外资2.1亿美元，分别增长25%和20.2%。实现外贸进出口总额4.6亿美元，增长40.9%。徽州经济开发区成为全市首家百亿产值园区。
        **（六）社会事业全面进步。**我市被评为国家科技进步先进市，永新股份跻身中国创新型企业百强。城乡义务教育经费保障提标扩面，加固和改造中小学校舍9.75万平方米，新改扩建幼儿园31所。黄山职业技术学院加快建设，歙县行知学校、黄山旅游管理学校跻身国家中职改革发展示范学校。完成35个乡镇综合文化站、337个农家书屋建设。“新安源有机茶”荣获全市茶叶企业首个中国驰名商标，4家企业被评为中华老字号，渔梁老街入选中国历史文化名街，屯溪老街跻身全国首批生态（社区）博物馆示范点。政府举办的基层医疗机构100%实行药品零差率销售，新型农村合作医疗参合率达99.3%。继续保持低生育水平，人口自然增长率控制在4‰以下。圆满完成第八届村委会换届选举工作，城市和谐社区建设扎实推进。强化食品药品安全监管，深入开展“安全生产年”活动。加强和创新社会管理，启动实施“六五”普法，深入开展矛盾纠纷排查化解，着力解决企业改制等历史遗留问题，全市信访形势总体平稳。加强社会治安防控体系建设，严厉打击各类违法犯罪活动，社会保持和谐稳定。国防动员、民兵预备役、人民防空和双拥优抚安置工作不断加强。妇女、未成年人、老年人、残疾人权益得到较好保障。新闻出版、地方志、文学艺术、哲学社会科学以及统计、档案、粮食、农机、气象、地质、地震和民族、宗教、侨务、对台等工作取得新的成绩。
        **（七）自身建设不断加强。**深入开展创先争优和机关作风集中整治活动，继续加强效能、政风、行风“三位一体”考核评议，办好“政风行风热线”和“市民连线”，受理群众和企业来信来电5900件。积极开展依法行政特色示范创建活动，深化行政审批制度改革，扎实推进政府信息公开和政务公开，市行政服务中心全年受理各类行政审批及服务事项14.27万件，提前办结率达99.1％。自觉接受市人大及其常委会的法律监督、工作监督和市政协的民主监督，进一步密切与各民主党派、工会、共青团、妇联等人民团体的联系，完善重大问题集体决策、专家咨询、社会公示和听证制度，认真听取各方面的意见和建议，推进决策科学化、民主化、法制化，市政府系统承办的423件人大代表建议、议案和政协提案全部办结。规范政府招投标管理，中心城区建设工程招投标、市直政府采购、国有产权出让、土地使用权出让业务相继纳入市公共资源交易平台，全年累计进场交易总额38.68亿元。严格落实党风廉政建设责任制和《廉政准则》各项规定，扎实开展工程建设领域专项治理，加大商业贿赂和损害群众利益不正之风等治理力度，切实解决损害群众利益的突出问题，严肃查处了一批违法违纪案件。
        各位代表！
        回顾过去的一年，发展令人振奋，成绩来之不易！这是省委、省政府和市委正确领导的结果，是市人大、市政协有力监督、大力支持的结果，是全市上下凝心聚力、顽强拼搏的结果。在此，我代表市人民政府，向全市广大工人、农民、知识分子、干部和离退休老同志，向驻黄解放军指战员、武警官兵和政法干警，向各民主党派、各人民团体和各界人士，致以崇高的敬意！向关心、支持黄山现代化建设的中央、省驻黄单位、海内外友好人士和外来投资者、创业者，表示衷心的感谢！
        在充分肯定成绩的同时，我们也清醒地看到，经济社会发展中还存在不少困难和问题。主要是：经济实力不强，特别是作为支柱产业的服务业结构单一、提速不快；节能减排任务艰巨，经济发展与生态环境保护面临双重压力；城乡发展不均衡，部分深山区、库区群众生活还很困难；政府职能需要进一步转变，领导和推动科学发展、建设现代国际旅游城市的能力需要进一步提升。对此，我们一定高度重视，采取更加有力的措施认真加以解决。

**二、2012年主要工作**

        今年是实施“十二五”规划承上启下的重要一年，是全面完成本届政府任期目标的收官之年。因此，做好今年的各项工作，具有十分重大的意义。需要看到，当前，一方面，国内外经济形势严峻复杂，经济增长下行压力加大，我们必须始终保持清醒的头脑，切实增强忧患意识，做好应对各种困难、迎接更大挑战的准备。另一方面，国家将继续实施积极的财政政策和稳健的货币政策，为我们加快发展提供了有力保障；我市仍处在不可多得的重要战略机遇期和大有作为的黄金发展期，特别是国家服务业综合改革试点、新安江流域生态补偿机制试点和徽州文化生态保护区、皖南国际旅游文化示范区四大战略平台的构建，为我们加快发展提供了有力支撑。我们一定要振奋精神、坚定信心，趋利避害、攻坚克难，奋力实现跨越发展。
        根据市委五届二次全会精神，今年政府工作的总体要求是：以邓小平理论和“三个代表”重要思想为指导，深入贯彻落实科学发展观，全面贯彻党的十七届六中全会、中央经济工作会议和省第九次党代会、省经济工作会议精神，按照市第五次党代会和市委五届二次全会的总体部署，紧紧围绕建设现代国际旅游城市的战略目标，以科学发展为主题，以全面转型、加速崛起、富民强市为主线，牢牢把握稳中求进的工作总基调，持续推动转型发展、开放发展、绿色发展、和谐发展，继续推进“十大工程”和“四区”建设，全面掀起招商引资、项目推进、旅游营销、绿色提升、重点村镇环境风貌整治和扶贫开发“六个高潮”，着力稳定经济增长，着力调整经济结构，着力深化改革开放，着力保障改善民生，保持经济又好又快发展，保持社会大局和谐稳定，以优异成绩迎接党的十八大胜利召开。
        综合考虑，今年经济社会发展的主要预期目标是：地区生产总值增长12%；财政收入增长20%以上；规上工业增加值增长25%以上；固定资产投资增长20%以上；社会消费品零售总额增长17%；居民消费价格涨幅控制在4%左右；城镇居民人均可支配收入增长15%，农民人均纯收入增长16%；万元生产总值能耗、主要污染物排放总量控制在省政府下达目标以内。在实际执行中，要在提升质量和效益的前提下，千方百计发展得更快一些。
        重点做好以下十个方面工作：
        **（一）全力扩大有效投入**
        全面掀起招商引资、项目推进高潮，进一步强化经济社会发展的主动力。
        **强力推进项目建设。**突出抓好600个“十大工程”重点项目建设，确保完成投资460亿元。全力推进京福高铁工程，开工建设国道205线黄山段示范改造、省道324线昱岭关至大阜改建工程，全面完成黄祁高速公路建设和省道218线甘棠至焦村改建，力争济祁高速殷汇至祁门段、歙县界牌岭至黟县渔亭一级公路、黄山机场4D级改造二期工程开工建设。继续做好黄杭高铁、皖赣铁路扩能改造、黄千高速公路等一批重大项目前期工作，力争月潭水库工程项目获国家批准立项，全年争取国债资金超过4亿元，重大项目储备超400个、总投资超4000亿元。进一步落实项目推进工作机制，确保新开工亿元以上项目100个以上，建成竣工亿元以上项目100个以上。
        **大力开展招商引资。**围绕工业优势产业、战略性新兴产业和旅游、文化、生态优势资源，系列推介400个、总投资近1900亿元的重点项目。进一步落实重点部门招商责任制，形成信息共享、上下联动、横向互动的招商新格局。切实发挥企业主体作用，选择100家重点企业开展以商引商、委托招商。精心策划“百村千幢”文化产业、徽州文化艺术长廊、高铁新区、黄山经济开发区等系列专题招商活动，积极参与徽商大会、中博会等重大招商活动，继续抓好与40家以上央企、20家以上省属大企业、500家以上全国知名民企和40家以上港澳台企业及世界500强企业的对接，确保全年引进内资突破470亿元，实际利用外资2.4亿美元，新签亿元以上重大项目100个以上、总投资超400亿元。
        **切实抓好融资保障。**持续推进金融发展与服务，力争银企对接承诺贷款达到100亿元，承诺贷款兑现率达到100%。全年新增贷款110亿元（全口径），贷款增幅高于全国、全省平均水平，小型微型企业贷款增速超过全市平均水平。积极引进市外商业银行在我市设立分支机构，规范发展小额贷款公司和融资性担保公司，力争农村银行实现区县全覆盖。启动实施世行贷款新农村建设（百村）示范项目。加强政府融资平台建设，力争全市平台融资达到100亿元，市城投公司融资超过20亿元，并力争二期企业债券上市发行。
        **（二）大力推进服务业发展**
        坚持旅游、文化、生态“三位一体”发展，全面掀起旅游营销高潮，加快打造和形成“一三五”特色发展格局。
        **扎实开展服务业综合改革试点。**认真贯彻落实全省服务业发展大会精神，强力推进服务业创新发展，确保实现增加值180亿元，增长12%。实施服务业重大建设项目142个，完成投资150亿元。打响徽韵、元一大型歌舞、花山谜窟激光秀、宏村阿菊等大型演艺产品，打造服务业发展的新亮点。全面完成市服务业发展总体规划和各专项规划编制工作，全力开展徽州文化艺术长廊、宏村、屯溪老街服务业集聚区以及太平湖旅游度假区、歙县滨江服务业长廊、万安旅游文化产业集聚区建设。继续扩大黄山口岸对外开放，巩固黄山至首尔、台北以及天津、西安、厦门等境内外航线，力争开通更多国内国际航班航线。认真落实省政府加快发展服务业若干政策，用好服务业发展专项资金，支持服务业企业做大做强。健全服务业统计指标体系，完善考核办法。
        **推进旅游业快速发展。**围绕打响“天地之美、美在黄山，人生有梦、梦圆徽州”的城市形象主题口号，实施“黄山欢乐健康游”活动，完善激励措施，创新促销方式，力争旅游接待量突破3500万人次，增长15%，旅游总收入突破300亿元，增长22%；其中，入境游客突破160万人次，增长22%，旅游创汇4.8亿美元，增长26%。深入推进皖南国际旅游文化示范区建设。精心办好第十六届黄山国际旅游节暨徽文化节、世界武术大会、国际山地车节等节庆赛事。加快黄山国际精品旅游景区打造，西海大峡谷观光缆车建成运营，力争世界遗产地可持续发展观测区、全球目的地可持续旅游标准实验区授牌。大力推进旅游重要节点建设，加快太平湖、徽州古城和棠樾牌坊群、呈坎争创国家5A级景区步伐，加大齐云山养生旅游、牯牛降生态旅游开发力度。大力推广新安江滨水旅游，积极拓展户外运动，打造旅游新亮点。持续推进乡村旅游发展，深入实施中法乡村旅游合作示范项目。加大旅游市场主体培育力度，支持旅游企业做大做强。深入开展“四大整治”，切实抓好旅游自驾车营地、停车场、标识标牌等配套设施建设，启动国家旅游应急救援黄山队建设，建成“智慧黄山”旅游调度指挥中心。
        加快发展其它服务业。繁荣城乡消费市场，营造良好消费环境，全年实现社会消费品零售总额173亿元。加快大型购物中心、大型批发市场和专业市场建设，沃尔玛、梅西百货建成营业，创建省级以上商业示范社区1～2 个。加强房地产市场分类管理，保持房地产市场健康发展。推进雨润国际会议中心等重大项目建设，精心培育商务会展市场。加大农超对接力度，扎实开展社区“双进”、“万村千乡”市场工程。大力发展徽菜产业，建成中国徽菜博物馆。推动社区、家政、中介等服务业发展。抓住国家和省扶持物流业发展优惠政策，加速物流业健康发展，构筑区域商贸物流基地。
        **（三）加快新型工业化进程**
        按照“三个围绕、一个不上”和“三个集中”的要求，进一步做大做强工业经济，坚定不移地走具有黄山特色的新型工业化道路。
        **全面提速工业经济。**组织实施优势产业和大企业大集团培育行动计划，继续安排市优势产业发展专项资金5000万元，加大机械电子等七大优势产业发展力度，着力抓好永佳集团、金马股份、皖南机床、黄汽电、中显微电子、联强纺织、恒远等40户优势产业龙头企业的提速升级，加快建设电子信息产业基地、装备制造业基地、汽摩配产业基地，力争全年新增规上企业50户以上，产值超5亿元企业突破10户，七大优势产业实现产值400亿元、百亿元产业超过2个。大力发展战略性新兴产业，加快培育新材料、高端装备制造、节能环保、新能源等一批新兴产业集群，抓好40个市级战略性新兴产业引导资金补助项目建设。加强对重点行业、重点企业的运行监测和协调服务，加快企业上市步伐。着力培育一批特色鲜明、竞争力强的进出口企业和产业基地，确保外贸进出口总额增长20%。
        **强力推进园区建设。**组织实施百亿园区培育行动计划，全力推进182个园区提速增效工程重点项目建设，着力提升园区招商项目质量，确保新签亿元以上工业项目40个以上，竣工项目30个以上，完成投资80亿元以上，园区工业占全市工业的比重超过70%。加快市循环经济园建设，支持九龙低碳园区申报省级开发区，歙县经济开发区成为百亿产值园区。全力推进市经济开发区“六园三片一线”建设，确保新签约、新开工、新投产亿元以上项目各10个以上，建成工业产值超5亿元企业1家、超3亿元企业2家、超亿元企业10家，完成固定资产投资35亿元，实现工业总产值35亿元，争创国家级低碳高新经济开发区。
        **着力强化科技创新。**扎实开展国家技术创新工程试点，组织实施技术改造百千工程，突出抓好226个投资超千万元的重点技改项目建设，完成投资50亿元以上，新增创新型（试点）企业10家、国家高新技术企业6家，新建省级以上高新技术产业基地2个，绿色软包装新材料高新技术产业基地跻身国家级产业基地行列，省机床工具高新技术产业基地正式运行。
        **（四）扎实抓好“三农”工作**
        坚持“三化”同步，加大农业科技创新力度，全面掀起扶贫开发高潮，促进农业增效、农民增收、农村发展。
        **强力推进扶贫开发。**把稳定解决扶贫对象温饱、实现脱贫致富作为首要任务，认真贯彻落实农村扶贫开发纲要，制定实施全市新时期扶贫规划，落实新的扶贫标准和范围，确保8000人稳定脱贫。市财政投入500万元，设立市级扶贫开发专项资金，整合各类涉农专项资金，进一步加大扶贫开发投入力度。大力实施社会扶贫，整合调动机关、企事业单位等各方力量参与扶贫开发。深入实施整村推进扶贫工程和产业扶贫，加大农村贫困劳动力培训转移力度，增强贫困地区自我发展能力，确保贫困地区农民人均纯收入增长高于全国、全省平均水平。
        **加快发展现代农业。**围绕特色主导产业，大力推进农业产业化，力争产值超亿元龙头企业达到15家以上，规模以上农产品加工业产值达到138亿元。深入实施生态茶园“双十”工程，新建高效生态茶园5万亩，办好黄山茶叶暨名优农产品推介会，确保茶业综合产值超过70亿元。抓好黄山区、徽州区2个省级现代农业示范区建设。扎实推进林业产业化“4646”工程，大力发展油茶、毛竹等特色经济林，林产业总产值达到130亿元。深入推进集体林权制度配套改革，实施黄山区全国国有林场改革试点。积极开展政策性农业保险。加强基层农技推广体系建设，深入实施科技进村入户工程，大力推广先进实用技术，积极发展各类科技示范户、示范片。切实抓好动植物疫病防控，建成国家茶叶检验中心，争取实施农产品质量安全检验检测中心项目。
        **深入推进新农村建设。**加强农村公路建设，完成农村危桥改造，实现全市所有建制村通水泥（油）路目标。加快病险水库除险加固和农田水利兴修，抓好黄山区、歙县、休宁县３个小农水重点县和祁门县小农水专项工程建设。加快农村电网升级改造。新建农村户用沼气池500口。再解决10万农村人口饮水安全问题。加强农村公共卫生服务体系建设，新型农村合作医疗参合率保持在95%以上。规范实施村级公益事业建设“一事一议”财政奖补试点。打造一批新农村建设模范村，新增“十有”达标村、基本达标村各50个。
        **（五）强力推进城镇建设**
        以建设重要的区域中心城市为目标，加速推进重要节点建设，全面掀起重点村镇环境风貌整治高潮，加快形成现代国际旅游城市新亮点。
        **突出抓好中心城区建设。**完成建设投资140亿元，增长30%以上。加快完善新安江延伸段东区道路、绿化、亮化和配套市政设施，全面建成新安江延伸段综合开发工程。启动新安江上游综合治理工程，开工横江桥至梅林大桥段开发治理项目，配套实施两岸市政基础设施和齐云大道两侧市政交通设施建设。建成花山大桥，续建文峰桥。新建续建仙人洞南路、新园东路、梅林南路等道路。新开工和竣工高层建筑各100幢以上。实施江心洲文化体育广场建设工程，改造提升世纪广场、昱中花园等9个公园广场。继续实施“三小”改造工程，建成城市展示馆、图书馆、档案馆、老年大学和老干部活动中心、残疾人服务中心等公益性项目。全力推进高铁新区站前广场、高品质居住区等五大板块建设，完成投资30亿元。新开工和续建徽州文化艺术长廊主要工程项目13个以上，百师宫等8个项目以及优先建设区全面建成并部分投入运营。全面完成各类保障性住房建设任务。加快总建筑面积68.4万平方米、总套数5204套的20个安置区建设。
        **加大区县城和小城镇建设力度。**全市完成城镇建设投资310亿元，城镇化率提高1.5个百分点。突出抓好屯溪区江南新城、黄山区城市道路及附属设施建设、徽州区新四军军部旧址及周边环境整治二期、歙县丰乐河整治及城区二环路建设、休宁县横江海阳段综合整治、黟县漳河沿岸整治及综合开发、祁门县新城区建设等项目，完成区县城建设投资120亿元。深化扩权强镇改革，抓好汤口新城、宏村古村落保护开发等城镇建设项目，46个建制镇完成投资73.3亿元。以市域范围内高速公路、铁路、主要旅游干线、城际交通干线两侧300米可视范围和重要景区景点周边为重点，依法开展控违拆违、改徽建徽、环境整治三大行动，用3到5年时间完成1万幢、170万平方米非徽派建筑整治改徽工作。
        **提升城市管理和创建水平。**强化规划龙头地位，进一步完善重点区域控制性详规和各类专项规划，并确保规划严格执行。打造黄山路、滨江路等6条户外广告管理样板路，实施旅游公厕改造升级和环卫基础设施配套提升工程，新建建筑垃圾处置场，试点生活垃圾分类收集。加大市容环境综合整治，着力解决摊点乱摆、车辆乱停、违法乱建、占道乱放、垃圾乱倒、渣土乱撒以及小区物业管理等方面突出问题。积极开展多种形式的文明创建活动，争创全国文明城市。
        **（六）加大生态建设和环境保护力度**
        全面掀起绿色提升高潮，全力保护绿水青山，积极争创联合国人居环境奖城市。
        **深入实施新安江流域综合治理。**抢抓国家编制和实施千岛湖及新安江流域水环境保护与生态建设总体规划等重大机遇，推进一批综合治理重点项目，并以新安江流域生态补偿机制为支撑，争取国家更大的支持。抓好国家开发银行专项贷款的争取和实施。启动新安江流域水源涵养林工程。开工建设新安江水质监测中心、流域水管理平台项目，实施市污水处理厂二期及其管网配套工程。抓好新安江流域企业转型升级工程，搬迁20户、关停10户、优化升级40户。加大沿线水土保持和污染治理力度，积极争创国家级水生态系统保护与修复试点市。
        **继续抓好生态建设。**投资3.1亿元，建成绿色质量提升点360个，建设绿化苗木基地2万亩，打造市级示范基地20个；完成新安江延伸段130万平方米绿化提升。扎实推进黄山生物多样性保护工程。抓好松材线虫病三道防线、世行贷款林业综合发展等生态工程项目建设，完成新造林10万亩。加强森林防火工作。强化河道采砂和集中供水水源地管理。加快推进歙县金竹岭、休宁阳台等重点地质灾害点防治。争创省级以上生态乡镇、生态村各5个，创建全市首批优质生态村30个。
        **扎实抓好节能减排。**严格政府目标责任考核，完善工作推进机制。以4项主要污染物减排为抓手，强化国控、省控和减排项目监管，加大涉铅等重金属排放企业、化工和造纸行业、工业园区、尾矿库整治力度，加强达限机动车强制报废工作。开展河道湖汊网箱养鱼和沿岸规模化畜禽养殖场污染综合整治。争取再有20个乡镇列入省农村清洁工程，61个乡镇533个村分别建成垃圾集中处置和收集转运系统，全市城镇生活垃圾无害化处理率达90%以上。抓好年耗能3000吨标煤以上企业和列入省重点监控范围企业的节能工作。加强交通运输和公共机构节能监管。大力推行太阳能建筑一体化应用，积极申报全国再生资源回收体系建设试点城市。坚持依法依规和节约集约用地，加快闲置土地清理，探索解决建设用地的路径和措施。
        **（七）推动文化大发展大繁荣**
        坚持传承与创新并举，大力发展文化产业和文化事业，全面提升黄山文化软实力。
        **加强徽文化资源保护利用。**继续推进徽州文化生态保护区建设。强力实施“百村千幢”古民居保护利用工程，提前一年全面完成古民居保护利用目标任务。加大文化遗产保护力度，力争新增全国重点文物保护单位5处、省级重点文物保护单位30处。做好徽州历史档案征集抢救工作。着力推进非遗保护传承利用，积极申办中国黄山非物质文化遗产传统技艺大展，争创国家级非遗传承基地。
        **大力发展文化产业。**继续实施文化产业精品打造工程，力争文化产业增加值增长30%。加快推进百佳摄影点、屯溪老街整体改造提升、徽州古城保护与开发等一批重点文化项目建设。充分发挥徽州文化艺术长廊的龙头和集聚作用，做大做强竹艺轩、翰林堂等一批骨干文化企业，推进徽州四雕、文房四宝、万安罗盘、徽州漆器等规模化生产。大力发展影视摄影、写生书画等新兴产业，加快秀里影视村二期和呈坎影视写生基地建设，继续举办黄山、黟县国际摄影大展，积极申报国家级影视产业基地。新建改扩建一批特色博物馆，做特做活文博产业。大力发展红色文化、知青文化、休闲文化、民俗文化和宗教文化，不断丰富文化业态。
        **着力提升公共文化服务水平。**推进区县文体科普中心、数字影院、图书馆建设，进一步完善乡镇综合文化站、农家书屋和社区文化基础设施，举办第六届徽州读书节，积极开展送戏下乡进社区和文化广场活动。大力弘扬民俗民间文化，切实抓好徽州文化研究、文艺创作和哲学社会科学事业。完成540个自然村广播电视“村村通”任务，继续实施好农村电影放映工程。加强文化市场管理，加大网吧、娱乐场所整治，净化校园周边文化环境。
        **（八）不断改善民计民生**
        始终坚持以人为本，着力保障和改善民生，加快推进基本公共服务均等化，让广大人民群众共享改革发展成果。
        **扎实抓好民生工程。**认真组织实施省定33项、市定3项民生工程，进一步加大资金投入，确保全市新增财力优先用于民生工程建设。强化民生工程资金和质量监管，完善长效机制，巩固建设成果，确保民生工程发挥持久效益，着力打造群众满意工程，确保全市民生工程工作继续走在全省前列。
        **加强就业和保障工作。**坚持就业优先战略，努力扩大就业规模。统筹抓好高校毕业生、城镇就业困难人员等重点人群就业。实施创业扶持工程，促进以创业带动就业，积极争创省级创业型城市。全年新增城镇就业1.8万人，城镇登记失业率控制在4.1%以内。加大社会保险扩面征缴力度，进一步提高城乡居民社会养老保险参保率。推进生育保险市级统筹。加快发放社会保障卡。做好城乡低保和农村五保工作，提高城乡医疗救助水平。不断完善社会救助和保障标准与物价上涨挂钩联动机制。
        **大力发展社会事业。**加大财政对教育的投入。新建和改扩建22所公办幼儿园，抓好35所义务教育学校标准化建设和11所学校宿舍楼建设，完成中小学校舍安全工程1.7万平方米。谋划和启动在新城区建设2所中小学校，新建和改造提升一批示范中小学，适度扩大省级示范高中办学规模。组织实施教育信息化工程和特殊教育关爱工程。支持重点职业学校发展，黄山职业技术学院正式对外招生。支持区县争创新一轮全国科技进步先进县。加强科普宣传工作，提高全民科学素质。积极培育和引进高层次、高技能人才，加强人才队伍建设。稳步推进事业单位分类改革。巩固完善基层医药卫生体制综合改革。扎实开展休宁县省公立医院改革试点。异地迁建市二院，完成市中医院、市经济开发区首康医院和歙县、休宁、祁门、徽州区医院建设。深入开展爱国卫生运动。夯实人口和计划生育基层基础工作，加强城市和流动人口计生服务管理，继续稳定低生育水平，人口自然增长率控制在4.5‰以内。加快构建全民健身体系，积极备战省十三运。加强国防教育、国防动员和后备力量建设，推动军民融合式发展，争创新一轮全国双拥模范城。
        **（九）加强和创新社会管理**
        认真落实党中央国务院关于加强和创新社会管理的重大战略部署，让城乡广大群众过上更加幸福安宁的生活。
        **夯实社会管理基层基础。**推进社区综合体制改革，调整优化布局，加大财政投入，提高保障水平，推动社区管理服务良性运行。深化村务公开和民主管理规范化建设，巩固村级组织换届成果。继续开展“六五”普法，深入推进基层民主法制建设。坚持和完善干部驻点帮扶、结对共建、“驻村夜访”、“民情交流”、“三四五”群众工作法等好的制度和做法，着力解决影响社会和谐稳定的源头性、基础性、根本性问题。
        **强化公共安全体系建设。**进一步落实安全生产责任制，继续组织实施“8+1”工程和“6100”行动计划，构建安全生产长效机制。强化政府监管职责，落实企业主体责任，进一步加强重点行业、重点领域、重点企业的安全监管工作。深入开展中小学校、公众聚集场所、道路交通、旅游、矿山、化工、烟花爆竹、建筑等安全生产专项整治活动，消除各类隐患，坚决杜绝各类重特大安全事故发生。完善监管体制，强化监管责任，综合整治食品药品安全问题，全面提高食品药品安全保障水平。积极推动应急救援体系建设，提高应对自然灾害、事故灾难、公共卫生事件、社会安全事件的快速反应能力。
        **切实维护社会和谐稳定。**继续抓好用群众工作统揽信访工作试点，健全和完善接访下访工作机制，建立市级联合接访中心和“信、访、网、电”多位一体的综合受理平台，畅通社情民意反映渠道。加大矛盾纠纷排查调处力度，构建人民调解、行政调解、司法调解相结合的“大调解”工作格局。实现社会稳定风险评估工作机制建设全覆盖，妥善解决土地和房屋征收、医患纠纷等引发的各类矛盾，掌握网络动态，疏导社会情绪，最大限度地消除不稳定因素。不断完善社会治安防控体系建设，严厉打击各类违法犯罪活动，确保实现创建全国社会治安综合治理优秀市“三连冠”、捧回“长安杯”。创新实有人口动态管理服务、闲散青少年群体教育帮助和预防犯罪、刑释解教人员安置帮教和社区矫正工作，为人民群众营造安居乐业的社会环境。
        **（十）不断加强政府自身建设**
        牢固树立执政为民理念，不断提高政府系统执行力和服务水平，全面适应新形势和新任务的要求。
        **强化执行力，狠抓工作落实。**深入开展机关效能和作风建设，强化行政问责，确保政令畅通，实现政府工作全面提速。加强对加快转变经济发展方式和重大决策部署监督检查，加强对重点工作、重大项目的考核考评，确保各项工作不折不扣落实到位。深入开展创先争优活动，始终保持昂扬向上的精神状态。继续办好“政风行风热线”和“市民连线”，继续开展效能、政风、行风“三位一体”民主评议工作，百家企业评机关、百企千户评窗口、民主评议基层站所活动，确保机关运转更为规范协调、工作更加有序高效。
        **强化公信力，坚持依法行政。**牢固树立社会主义法治理念，把政府建设成为彰显法治精神、促进社会公平正义的行政机关。不断加强政府法制工作，深入开展依法行政特色示范创建活动。全面提升行政执法队伍素质，提高行政执法水平。进一步加强行政服务中心建设，促进政务服务均等化、规范化、高效化。认真贯彻实施政府信息公开条例，全面推进政务公开和办事公开。自觉接受人大及其常委会的法律监督、工作监督和政协的民主监督，办好人大代表议案、建议和政协提案，及时向人大报告、向政协通报重大事项，认真落实人大的决议决定和审议意见，注重采纳政协的调研成果和意见建议。广泛听取民主党派、工商联、无党派人士和各人民团体的意见，主动接受社会公众和新闻舆论监督。
        **强化约束力，加强廉政建设。**认真落实《廉政准则》，健全权力运行制约和监督机制，用制度管权、按制度办事、靠制度管人，从源头上预防和治理腐败。切实加强廉政建设，全面开展廉政风险防控管理，加快推进电子政务和“数字监察”系统建设，规范项目招投标、国有土地出让、产权交易、政府采购、政府投资、公务消费等行为。深入开展反腐败斗争，充分发挥监察、审计部门的监督作用，加强对扶贫救济、社保基金等社会公共性资金的监管。积极推进节约型机关建设，勤俭办一切事业。

        各位代表！
        发展成就令人鼓舞，新的任务催人奋进！让我们在省委、省政府和市委的坚强领导下，高举中国特色社会主义伟大旗帜，深入贯彻落实科学发展观，依靠全市人民的智慧和力量，坚定信心，扎实工作，拼搏奋进，全面完成今年的各项目标任务，加快建设现代国际旅游城市，以优异成绩迎接党的十八大胜利召开！
